# The causes of negative countertransference in its cultural aspect among psychiatric residents in Tunisia

**DOI:** 10.1192/j.eurpsy.2024.560

**Published:** 2024-08-27

**Authors:** D. Mezri, S. Walha, S. Hamzaoui, K. Mahfoudh, A. Ouertani, U. Ouali, A. Aissa, R. Jomli

**Affiliations:** Avicenne, Razi hospital, Manouba, Tunisia

## Abstract

**Introduction:**

Negative countertransference in psychiatry refers to the therapist’s unfavorable emotional reactions to the patient, such as anger and frustration, which can hinder the therapeutic relationship and the client’s progress. This is why it is imperative to study the causes of this negative counter-transference, such as cultural causes, to ensure effective treatment, appropriate care and better comfort for psychiatry residents during their professional practice.

**Objectives:**

To study the cultural causes of negative countertransference among psychiatric residents in Tunisia and their coping behavior.

**Methods:**

This cross-sectional study was carried out among Tunisian residents working in psychiatric departments, using a questionnaire deployed via Google Forms.

**Results:**

The study involved 26 residents with 23 females. The average age was 27.57 years with extremes ranging from 26 to 32. The participants were family doctors practicing in psychiatric wards (26.9%), first year psychiatry residents (15.4%), second year psychiatry residents (23.1%), third year psychiatry residents (19.2%), fourth year psychiatry residents (11.5%) and child psychiatry residents (3.8%). The majority of residents admitted having had a negative transference towards a patient (88.5%). The level of frustration felt by residents during this counter-transference on a scale of 100 varied from 1 to 100 with an average of 61.9. Substance abuse was the primary cause in 52.17% of cases. The second cause was the patient’s ideology, with a percentage equal to 43.47%. The same percentage of 17.39% was for traditions, socio-economic level and membership of a particular political group. In 82.6% of cases, residents tried to analyze this counter-transference and 65.2% of them managed to deal with their frustration. The feeling of guilt was experienced by 56.52% of practitioners and the same number of residents tried to avoid the patient. Among the participants, 43.47% discussed this difficulty with their supervising physician and only 4 residents asked to change patients.

**Conclusions:**

In conclusion, our study identified the cultural causes of negative countertransference in Tunisian psychiatry residents, including substance abuse, ideology, traditions, socio-economic level and politics. Understanding these causes is essential to resident training but also to the delivery of quality care in psychiatry. By integrating this knowledge into training, we can help residents recognize and manage negative countertransference, in order to improve the quality of care they provide to their patients.

**Disclosure of Interest:**

None Declared

